# Oxidative stress generated due to photocatalytic activity of biosynthesized selenium nanoparticles triggers cytoplasmic leakage leading to bacterial cell death

**DOI:** 10.1039/d2ra07827a

**Published:** 2023-04-12

**Authors:** Banishree Sahoo, Lipsa Leena Panigrahi, Sonali Jena, Suman Jha, Manoranjan Arakha

**Affiliations:** a Center for Biotechnology, Siksha ‘O’ Anusandhan (Deemed to be University) Bhubaneswar 751003 Odisha India marakha@soa.ac.in; b Department of Life Science, National Institute of Technology Rourkela Odisha 769008 India

## Abstract

The present work investigates the role of oxidative stress generated at biosynthesized selenium nanoparticles (SeNPs) interface in defining the antimicrobial and anti-biofilm activity. To this end, SeNPs with average size of 119 nm were synthesized rapidly during the growth of *Staphylococcus aureus* using the principle of green chemistry. The synthesis of SeNPs was confirmed by using different biophysical techniques like UV-vis spectroscopy, X-ray diffraction (XRD), field-emission scanning electron microscope (FE-SEM), EDX and zeta potential analysis. The obtained data from antimicrobial study revealed strong antimicrobial activity against both Gram-positive bacteria like *Bacillus subtilis* (MTCC 441) and Gram-negative bacteria like *Escherichia coli* (MTCC 443) and anti-biofilm activity against biofilm forming bacteria. The mechanism behind antimicrobial activity of biosynthesized SeNPs was explored by evaluating the amount of reactive oxygen species (ROS) generated at SeNPs interface due to photocatalytic activity. The experimental data obtained altogether concluded that, the ROS generated at SeNPs interface put stress on bacterial cell membrane causing leakage of cytoplasmic contents, leading to bacterial cell death.

## Abstract 2

The ROS generated at SeNPs interface put stress on bacterial cell membrane causing leakage of cytoplasmic contents leading to bacterial cell death.

## Graphical abstract

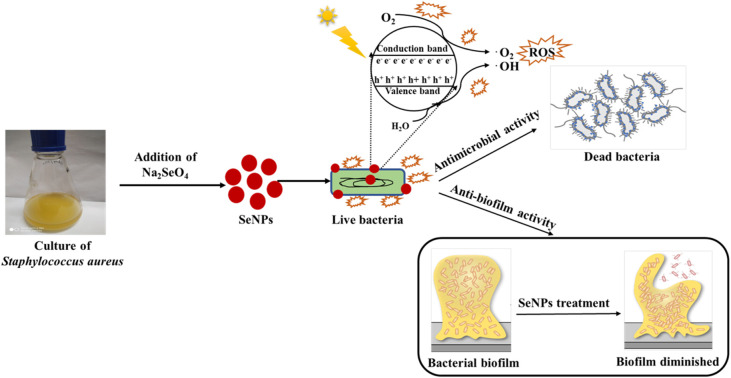

## 1. Introduction

In the prevailing environmental status quo, escalation of antibiotic resistant bacterial strains is becoming a threat to human health worldwide, since it is causing thousands of infections and deaths every year.^1^ Hence, scientists have been trying to formulate new and cost effective antibacterial formulations/strategies to control these antibiotic resistant bacterial strains. In addition, combating infections linked to biofilm formation is also considered as one of the major challenges to be overcome in this medical scientific arena. Bacteria, having the potential to form biofilm are encapsulated with a complex mixture of extracellular polymeric substances (EPS), which help them to acquire high tolerance capacity towards conventional antibiotics as well as classical host immune response.^2^ However, the two most challenging issues associated with biofilm infection are: biofilm formation on the host tissue and microbial colonisation on different abiotic surfaces such as medical devices.^2^ With increased use of medical devices in everyday life scenario, biofilm associated infections are emerging as major threat to the human health, approximately contributing up to 80% of total bacterial infections.^2^ Consequent lack in early diagnosis and correct targeting approach makes biofilm associated infectious diseases exorbitant and strategically problematic. Thus, making either removal of the colonized medical device in use or surgical debridement of the tissue infected with biofilm are ultimate alternatives. As such, researchers worldwide are trying to find out new anti-biofilm formulations to limit the bacterial growth and adhesion to various biotic and abiotic surfaces.^3^

In this context, researchers are now focusing on nanomaterials with antimicrobial properties as promising tools to limit bacterial growth and adhesion. Inside the biological environment, nanoparticles (NPs) of myriad shape and size interact with different biological macromolecules such as DNA, proteins, lipids, flavonoids, polysaccharide and form Bio-nano interface.^4^ Additionally, NPs possessing photocatalytic activity are capable of generating ROS upon interaction with biological entities resulting in cell death, hence could be used to control biofilm formation.^4,5^ NPs can inhibit the biochemical pathways of the bacterial cell leading to bacterial cell lysis resulting in non-viable bacterial cell. Among different NPs, metal/metal oxide NPs such as silver, zinc oxide, titanium dioxide *etc.* have excellent antimicrobial activity against both Gram-negative and Gram-positive bacteria, however toxicity is a major concern for various biological applications.^6,7^ Hence, selenium having significant antimicrobial activity and biocompatibility could be opted as alternative NPs for formulation of possible antimicrobial agents.^8^ Initially, Se was discovered by Jons Jacob Berzeliu in 1817, as by-product of sulphuric acid synthesis which can be found in different forms such as red coloured powder, black in vitreous form, and metallic grey in crystalline form.^9,10^ It has been reported that SeNPs possess unique properties in comparison to Se-containing compounds. Some of the attractive properties of SeNPs are: SeNPs possess selective cytotoxicity, it cause the death of cancer cells without exhibiting cytotoxic effect against normal cells.^11^ Additionally, SeNPs have the potential to exhibit cytotoxic effect even if at small concentrations.^11^

Although, number of biological agents available in nature such as plants and plant extracts, algae, bacteria and fungi can be employed for synthesis of NPs using the principle of green chemistry, however, synthesis of NPs using bacteria has drawn significant attention of researchers due to resistance of bacteria against different concentrations of heavy metals.^12–14^ The bacteria have the potential to survive at high metal ion concentrations due to various surviving skills of bacteria such as efflux systems, extracellular complexation, specific metal transport system *etc.*^12^ Hence, among various microorganisms, bacteria possess high adaptability to specific environmental conditions.^12^ Literatures have reported that, various microorganisms have the potential to resist selenium oxyanions such as selenite or selenate aerobically and reduce them into elemental selenium ion,^10^ hence could be used as potential candidate for synthesis of SeNPs using green synthesis methods.

In the present study we have determined the minimum inhibitory concentrations (MIC) of sodium selenate against *Staphylococcus aureus* (*S. aureus*), where the bacterial population anticipated to be equally populated with viable and non-viable cells. The study has optimized a novel green synthesis protocol for SeNPs synthesis using commonly available bacteria *S. aureus* at MIC concentration of sodium selenate. We hypothesized that; this method can be used for large scale production of SeNPs to fulfil the need of various nanotechnology industries. The formulated SeNPs were characterized using various biophysical techniques such as UV-visible spectroscope, XRD, zeta analyser, field emission scanning electron microscope (FE-SEM) *etc.* The antimicrobial activity of synthesized SeNPs was analysed using growth kinetic and CFU analysis studies, whereas anti-biofilm activity was analysed using Congo red agar plate method and microtiter plate assay. The mechanism behind antimicrobial activity was investigated by detecting amount of ROS generation at SeNPs interface and membrane leakage study. The data altogether demonstrated that the oxidative stress generated in the bacterial cells due to photocatalytic activity of SeNPs put stress on bacterial cell membrane leading to membrane leakage resulting in non-viable bacterial cells.

## 2. Experimental details

*S. aureus* (MTCC 96), *E. coli* (MTCC 443) and *B. subtilis* (MTCC 441) were purchased from Institute of Microbial Technology (IMTECH), Chandigarh, India. Sodium selenate anhydrous was purchased from Hi-Media Laboratories Pvt. Ltd, India. Nutrient agar and nutrient broth were purchased from SRL, Mumbai, India. 2′, 7′-Dichlorodihydrofluorescein diacetate (DCFH-DA) was obtained from Cayman chemicals, USA. All the above stated chemicals were analytical in grade and used for all experimental works without further purification.

### 2.1 Determination of minimum inhibitory concentration (MIC) of sodium selenate against *S. aureus*

Minimum inhibitory concentration (MIC) was evaluated to check the resistance of *S. aureus* bacteria against sodium selenate. The mother culture of the test bacteria was prepared by inoculating a loop full of bacteria into 10 mL of nutrient broth and incubated overnight at 37 °C and 120 rpm. Different reaction mixtures were prepared in test tubes taking different concentrations (0, 25, 50, 75, 100, 125 and 150 mM) of sodium selenate and 100 μL of bacterial culture. The volume of each reaction was adjusted to 3 mL by adding appropriate amount of nutrient broth. The bacterial growth was monitored by observing change in O.D. at 600 nm after 16 h of incubation using UV-vis spectrophotometer (Hitachi High-Tech in America).

### 2.2 Synthesis of selenium nanoparticles

For the synthesis of SeNPs, 5 mL of *S. aureus* mother culture was prepared by inoculating bacteria from slant into the nutrient broth and kept overnight in an incubator at 120 rpm and 37 °C. From the prepared culture, 1 mL of the bacterial culture was added to 100 mL of nutrient broth and incubated at 37 °C and 120 rpm agitation for the growth of the test bacteria. An appropriate amount of sodium selenate (MIC concentration, 75 mM) was added at the mid-log phase of the bacterial growth. Further, the reaction mixture was incubated for next 48 h at 37 °C and 120 rpm for SeNPs synthesis. Upon course of time, the colour of the reaction mixture was changed into ruby red, which indicated the synthesis of SeNPs. The characteristic ruby red colour is attributed to the Surface Plasmon Resonance (SPR) property of SeNPs.^15^ Then, the bacterial culture containing SeNPs was harvested by centrifugation at 6000 rpm for 30 min. The pellet was washed twice in distilled water and dried at 70 °C to obtain powder SeNPs.

### 2.3 Characterization of synthesized SeNPs

The XRD pattern of SeNPs was recorded on smart lab version Rigaku Xray Diffractometer (Tokyo, Japan), using Cu-Kα radiation, at a scan rate 20° min^−1^ with step size 0.05° over a 2*θ* range of 20 to 80. The X′-pert high score software having search and match facility was used to study different phases present in the synthesized SENPs sample. Morphological features like size and shape of the synthesized SeNPs was analysed with the help of FE-SEM (Nova Nano SEM 450, FEI company, Netherland). UV-Vis spectroscopy (HITACHI, Tokyo, Japan) was used to observe the surface plasmon resonance property of SeNPs. Moreover, EDX was used to analyse the elemental composition of synthesized SeNPs. The zeta analyser (Malvern Zetasizer Nano ZS90, Netherland) was used to evaluate the surface potential of synthesized SeNPs.

### 2.4 Antimicrobial activity of SeNPs

Initially, the antimicrobial activity of SeNPs against different Gram positive bacteria like *B. subtilis* and Gram-negative bacteria like *E. coli* was evaluated using different antimicrobial methods such as growth kinetics and colony forming unit (CFU) measurement *etc.* Briefly, a loop full of bacterial culture was taken from slant and inoculated into the nutrient broth and kept overnight at 37 °C and 120 rpm in a shaker incubator. Additionally, SeNPs stock solution was prepared by dispersing appropriate amount of SeNPs in deionised water and keeping it in sonicator for 20 min. The reaction mixtures for growth kinetic study were prepared in a 96 well plate in presence of different concentrations of SeNPs (25, 50, 100, 250, 500 μg mL^−1^) and reaction mixtures without SeNPs were treated as control. 20 μL of bacterial culture was added to different reaction mixtures and the volume was adjusted to 300 μL by adding appropriate amount of nutrient broth. Then, growth kinetic study was performed in a plate reader (Biorad imark plate reader) at regular intervals by measuring optical density (O.D) at 600 nm.

### 2.5 CFU count

The antimicrobial activity of synthesized SeNPs was further evaluated by estimating the number of viable bacterial cells upon SeNPs treatment. Briefly, 10 μL of bacterial samples were taken from the stationary phase of growth kinetics. Then, the samples were diluted 10 000 times in autoclaved distilled water and spread on the nutrient agar plate. The plates were incubated at 37 °C overnight. The colony forming units for each concentration of SeNPs was counted and compared with control to evaluate the viability of bacterial cells upon treatment with different concentrations of synthesized SeNPs.

### 2.6 Anti-biofilm activity of SeNPs

To evaluate the anti-biofilm activity of biosynthesized SeNPs, initially the biofilm producing ability of *S. aureus* bacterial strain was evaluated by culturing them on Congo red agar (CRA) plates. The media for CRA pate was prepared by taking Brain Heart Infusion broth (BHI), agar, sucrose, Congo red and distilled water following the protocol adopted by Mariana *et al.*^16^ Following autoclave, the plates were poured with media and solidified for further use. The mother culture of *S. aureus* was prepared by inoculating a loopful of bacterial strain into nutrient broth and kept at 37 °C for overnight in a shaker incubator. A loopful of bacteria from mother culture was streaked on solidified CRA agar plates and incubated at 37 °C for overnight. On the following day black colonies were observed which indicated the biofilm activity of the test bacteria. Further, to check the anti-biofilm activity of SeNPs against *S. aureus*, Congo red media was prepared and autoclaved. Different concentrations of biosynthesized SeNPs (100, 250 and 500 μg mL^−1^) was prepared followed by sonication and added to the media. The medium with SeNPs was poured to the respective plates and solidified. Then, *S. aureus* strain was streaked on the plates having different concentration of SeNPs and incubated at 37 °C to check the anti-biofilm activity of SeNPs.

Following visual observation on CRA plates, the anti-biofilm activity of different concentrations of SeNPs was also quantified by microtiter plate assay. For the same, *S. aureus* was cultured in 10 mL of luria broth and incubated at 37 °C to get bacteria culture up to 10^6^ CFU mL^−1^. 100 μL of *S. aureus* bacterial culture containing approximately 10^6^ CFU mL^−1^ of cells was added in 96-well plates with different concentrations of SeNPs (50, 100, 250, and 500 μg mL^−1^) and incubated overnight at 37 °C. Reaction mixtures without SeNPs treatment were taken as control. The biofilms formed were rinsed with 100 μL of PBS buffer to remove non adherent cells. After 10 min, the cells were replaced with 200 μL of 1% of crystal violet solution for 1 h followed by washing with PBS. Then the wells were dried and finally 200 μL of 95% methanol was added to resolubilize the biofilm and absorbance was taken at 595 nm using plate reader (Biorad imark plate reader).

### 2.7 Photocatalytic activity

The photocatalytic activity of SeNPs, biosynthesized by *S. aureus* was estimated by degradation of an organic dye, methylene blue (MB), under direct sunlight, following the protocol adopted by Joshi *et al.* with some modifications.^17^ Initially, a stock solution of MB dye was prepared by dissolving 10 mg of dye in 500 mL distilled water. Five milligram of synthesized SeNPs of various concentrations (100, 250 and 500 μg mL^−1^) were added to the MB dye solution. MB dye solution without SeNPs was treated as control. All the reaction mixtures were exposed to sunlight irradiation (temperature around 30–35 °C). The reactions were monitored using a UV-vis spectroscopy, observing absorbance at 660 nm at different time intervals like 0.5 and 2 h respectively.

### 2.8 ROS detection

To explore the effect of ROS generation due to photocatalytic activity of biosynthesized SeNPs on antimicrobial activity, ROS generated at SeNPs interface was evaluated using 2′, 7′-dichlorodihydrofluorescein diacetate (DCFH-DA). DCFH-DA is a peroxynitrite indicator used to investigate the generation of ROS upon SeNPs treatment.^18^ As a test organism, we have taken *E. coli* bacterial cells to evaluate the amount of ROS generation due to interaction of SeNPs. Hence, *E. coli* bacterial cells were cultured in a 96 well plate and treated with 200 μM of DCFH-DA. Different concentrations of SeNPs such as 10, 100 and 250 μg mL^−1^ was added to the reaction mixtures and fluorescence emission was observed at 523 nm with an excitation at 503 using Synergy H1 hybrid reader (Biotek, USA).

### 2.9 Analysis of cytoplasmic leakage

Upon treatment of bacterial cells with NPs, cytoplasmic materials, such as DNA and protein, are released from cells. Hence, in this study, we have taken an attempt to evaluate the cytoplasmic leakage of bacterial cells upon SeNPs treatment. For the estimation of DNA, *E. coli* (10 mL) was cultured in nutrient broth and kept for overnight. On the following day, the culture was harvested by centrifuging at 5000 rpm for 10 min. The pellet was washed and resuspended in PBS buffer (1×, pH 7.2). The number of the bacterial cells was adjusted to 1 × 10^5^ cells per mL. Different aliquots of cell suspensions were treated with SeNPs and incubated at room temperature for 3 and 5 h. The bacterial culture without SeNPs was treated as control. Then, the cultures were centrifuged at 5000 rpm for 10 min and the absorbance of supernatants was recorded at 260 nm.^19,20^

Additionally, for estimation of protein, *E. coli* bacterial culture was prepared by inoculating bacteria from slant culture into 30 mL of nutrient broth and incubated overnight in a shaker incubator. Then different concentrations of SeNPs (250, 500 μg mL^−1^) were added to different aliquots of bacterial cultures and incubated overnight at 37 °C with shaking at 120 rpm. After incubation, these cultures were centrifuged at 10 000 rpm for 30 min to remove the bacterial cell mass and the supernatant was collected and stored at 20 °C immediately. Control (without treatment of SeNPs) was treated as control. The concentration of protein leakage was determined by the Bradford assay.^18^

## 3. Results and discussion

### 3.1 MIC of sodium selenate against *S. aureus*

**Fig. 1:**
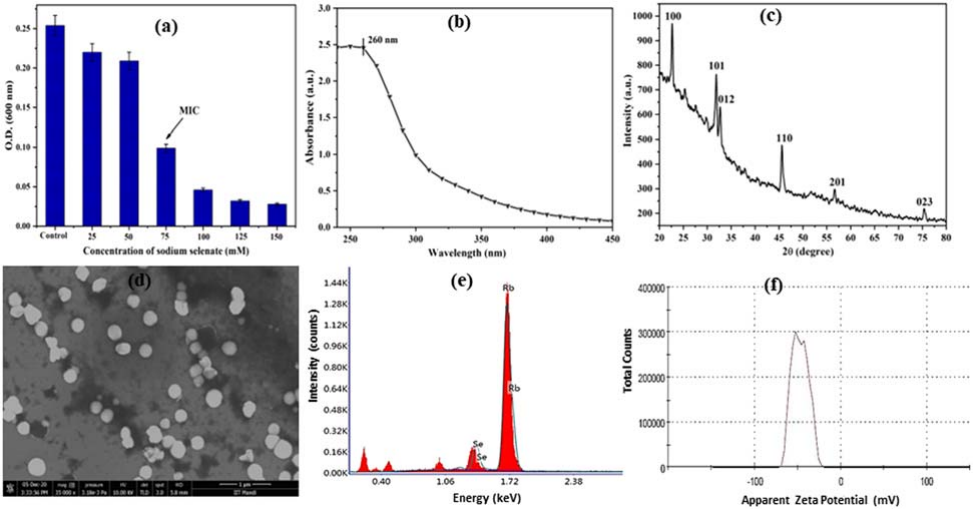
Characterization of biosynthesized SeNPs. (a) MIC of sodium selenate against *S. aureus*, (b) UV-vis absorption spectra of synthesized SeNPs, (c) XRD pattern of SeNPs, (d) FE-SEM image of SeNPs, (e) EDX spectra of SeNPs, (f) zeta potential analysis of SeNPs.

To check the viability of *S. aureus* in presence of different concentrations of sodium selenate, the MIC of sodium selenate was evaluated against *S. aureus*. From the experiment, it was observed that 75 mM is the MIC value of sodium selenate against *S. aureus*, where about 40% of viable cells were present in comparison to control (Fig. 1a). Additionally, for the synthesis of SeNPs, 75 mM sodium selenate (MIC concentration) was added to the *S. aureus* bacterial culture at mid log phase of the growth. The bacteria help in the synthesis of elemental SeNPs through the process of reduction of metal ions during their growth phase.

Generally, determination of MIC in this study indicates the toxicity level of sodium selenate against *S. aureus*. For the synthesis of NPs, microorganisms are used effectively as biological agents, hence considered as biological factories for the fabrication of various metal and metal oxide NPs. They have the ability to detoxify, accumulate and reduce heavy metal salts to metallic nanoparticles of narrow size distribution.^10^ Since few microorganism have the potential to resist selenium oxyanions such as selenite or selenate and reduce them into elemental selenium ion Se(0),^10^ hence we have chosen *S. aureus* as potential candidate for formulation of SeNPs. In this context, we have evaluated the MIC of sodium selenate against *S. aureus* and observed 75 mM to be MIC value, where bacterial population is equally populated with viable and non-viable cells. We hypothesized that the viable bacterial cells at MIC value will play vital role in the synthesis of SeNPs. As anticipated, upon incubation with sodium selenate (75 mM) the colour of the reaction mixture changed into ruby red, which confirmed the synthesis of SeNPs.

Mechanisms used by bacteria to reduce selenate leading to SeNPs synthesis, employing the selenite ion as a precursor, may include several metabolic pathways comprised of various enzymes and proteins. In this context, mechanism behind bacterial reduction of selenite and selenate to SeNPs generally involves different stages such as (i) transport of selenium oxyanions into bacterial cells (ii) redox reactions, (iii) elementary Se^0^ nuclei are exported out of the bacterial cell, and (iv) elementary Se^0^ are assembled to form SeNPs.^21^ The reduction of selenite to Se^0^ follows a series of reaction as mentioned below.^21^

1 Se^VI^O_4_^2−^ + 2e^−^ + 2H^+^ ⇆ Se^IV^O_3_^2−^ + H_2_O

2 Se^IV^O_3_^2−^ + 4e^−^ + 6H^+^ ⇆ Se^0^ + 3H_2_O

Microorganisms mediated NPs formulation is relatively faster and has drawn attention of various research groups because of their availability in nature, and possess the potential to synthesize various metal nanoparticles using extracellular and intracellular mechanism.^22^ Microbial extracts contain cofactors, enzymes, flavonoids, proteins, and terpenoids those serve as reducing and capping agents.^14^ The synthesized SeNPs can be functionalized by certain biomolecules such as proteins, carbohydrates, and lipids.^23^ Microorganisms produce elemental SeNPs with particular surface physico-chemical properties pertaining to the cellular moieties acting as capping agents. The major aim of taking *S. aureus* for synthesis of SeNPs is for the continuous production of NPs, *i.e.*, simultaneously bacterial growth and NP production, since it aspires to various NP-mediated industrial and theranostics applications.^14^

### 3.2 Characterization of biosynthesized SeNPs

Several protocols have been formulated for the synthesis of SeNPs, however, due to agglomeration propensity; maintenance of stability is the key issue in the synthesis of SeNPs. Hence, researchers are focusing on green synthesis methods to obtain well disperser and highly stable NPs. In this study, we have biosynthesized SeNPs from the bacteria *S. aureus*. Initially, the synthesized SeNPs were analysed using UV-Vis spectrophotometer. Upon synthesis of SeNPs, the colour of solution was observed to be ruby red, which is due to the Surface Plasmon Resonance (SPR) property of SeNPs. The absorption spectra of biosynthesized SeNPs were analysed between 200 and 700 nm using UV-Vis spectrophotometer and the characteristic absorption peak was found at 260 nm (Fig. 1b), which is very similar to the result reported by Gunti *et al.*^7^ The bandgap energy for SeNPs was determined using the equation *E*_bg_ = 1240/*λ*. Where *E*_bg_ is the band gap energy in eV and *λ* is the wavelength of light. From the equation, the calculated band gap energy for SeNPs was found to be 4.7 eV. In this context, Mellinas *et al.* have also synthesized SeNPs using *Theobroma cacao L.* bean shell extract and observed band gap energy to be 4.21 eV.^24^

The synthesis of SeNPs was further confirmed by X-ray diffraction spectroscopy as shown in Fig. 1c. The major diffraction peaks corresponding to different angle values such as 22.66° (100), 31.91° (101), 32.7° (012), 45.59° (110) and 75.38° (023) revealed that synthesized SeNPs has high purity and crystalline in nature. The 2*θ* value for peak (101) is 31.91°, which is the basic feature of SeNPs.^6^ As shown in the figure, d_101_ peak has the highest intensity inferring the dominance of d_101_ lattice plane in the synthesized SeNPs. Additionally, the obtained XRD spectra was analysed using X-pert high score software with search and match facility and found to be hexagonal in structure (JCPDS reference code-83-2439). The average particle size of SeNPs was calculated using Scherrer's equation.Particle size (*D*) = *Kλ*/*β* cos *θ*

where *λ* is the wavelength of X-ray (1.540 × 10^−10^ m), *K* is the proportionality coefficient, also referred as shape factor and its value is 0.9, *θ* is the Bragg angle and *β* is the full width at half maximum in radians. Using the above equation, the average particle size of SeNPs was calculated to be 119 nm. The morphological features of synthesized SeNPs were characterized using FE-SEM as shown in Fig. 1d. The synthesized SeNPs were found to be spherical in shape and well disperse. Additionally, Energy-dispersive X-ray spectroscopy (EDX) was used to evaluate the elemental composition of biosynthesized SeNPs. As shown in the Fig. 1e, the signal for selenium was observed, which confirmed the synthesis of SeNPs. The zeta potential measurement of SeNPs was illustrated in Fig. 1f which gives an idea about the stability of the SeNPs. The average surface potential of SeNPs was found to be −47.1 mV demonstrating well stability of SeNPs. In this context, Bhattacharjee *et al.* have also reported that nanoparticles having a zeta potential value of greater than 30 mV makes the colloidal system highly stable.^8^

### 3.3 Antimicrobial activity of SeNPs

**Fig. 2:**
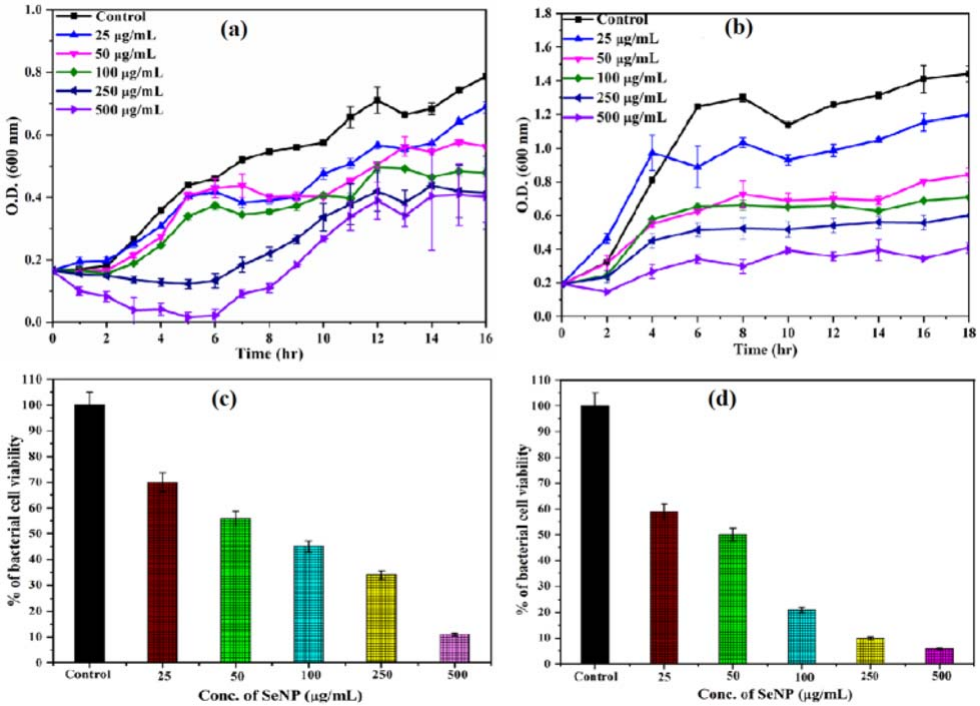
Growth kinetics of *B. subtilis* (a) and *E. coli* (b) in the absence and presence of different concentrations of SeNPs. Triplet experiments have been accomplished for every reaction and error bar was given to represent the standard error mean. Estimation of viable bacterial cells in presence of different concentrations of SeNPs. CFU of *B. subtilis* (c) and *E. coli* (d) cells were calculated as a percentage of viable cells in comparison to control.

Initially, the antimicrobial activity of biosynthesized SeNPs was evaluated by growth kinetics studies against Gram positive bacteria like *B. subtilis* and Gram-negative bacteria like *E. coli* at different concentrations of SeNPs (Fig. 2a and b). As shown in the figure, significant growth inhibition was observed in comparison to control upon addition of increasing concentrations of SeNPs. The antimicrobial activity of biosynthesized SeNPs was further investigated by CFU measurement studies (Fig. 2c and d). At lower concentration of SeNPs such as 25 μg mL^−1^, the percentage of viable bacterial cells was observed to be approximately 70% and 60% for *B. subtilis* (Fig. 2c) and *E. coli* (Fig. 2d) respectively. However, at higher concentration of SeNPs such as 250 μg mL^−1^, significant growth inhibition was observed for both bacteria, where the percentage of viable bacterial cells was observed to be around 30% and 10% in case of *B. subtilis* (Fig. 2c) and *E. coli* (Fig. 2d) respectively. The obtained data for different concentrations of SeNPs indicates that bacterial cell viability decreases with increase in concentration of SeNPs. The obtained results correlate to the results obtained from growth kinetic studies. The possible mechanism behind the antimicrobial activity of SeNPs has not been explored fully, hence needs to be investigated more. Among these bacterial strains, Gram-positive bacteria showed higher resistance to SeNPs in comparison to Gram-negative bacteria. Gram-positive bacteria have a thicker peptidoglycan layer about 20–80 nm, whereas Gram-negative bacteria have a thinner peptidoglycan layer of 10 nm.^25^ Hence, SeNPs can easily penetrate the cell wall of Gram-negative bacteria deforming the bacterial cell membrane resulting in non-viable cells. The disruption of the bacterial membrane occurred due to the abundance of ROS which leads to oxidative stress in bacterial cell and in turn, it triggers oxidation of proteins, lipids and DNA damage.^26^ Therefore, higher antimicrobial activity was observed for Gram-negative bacteria such as *E. coli* in comparison to Gram-positive bacteria such as *B. subtilis*. Various studies have demonstrated the antimicrobial activity of SeNPs.^27,28^ In this context, San Keskin *et al.* have reported that SeNPs has significant antimicrobial activity nearly at 1 mg mL^−1^ concentration against both *E. coli* and *S. aureus* with equal efficacy.^27^ However, in our study, we have observed bacterial growth inhibition significantly at lower concentration of SeNPs against both *B. subtilis* and *E. coli*.

### 3.4 Anti-biofilm activity of SeNPs

**Fig. 3:**
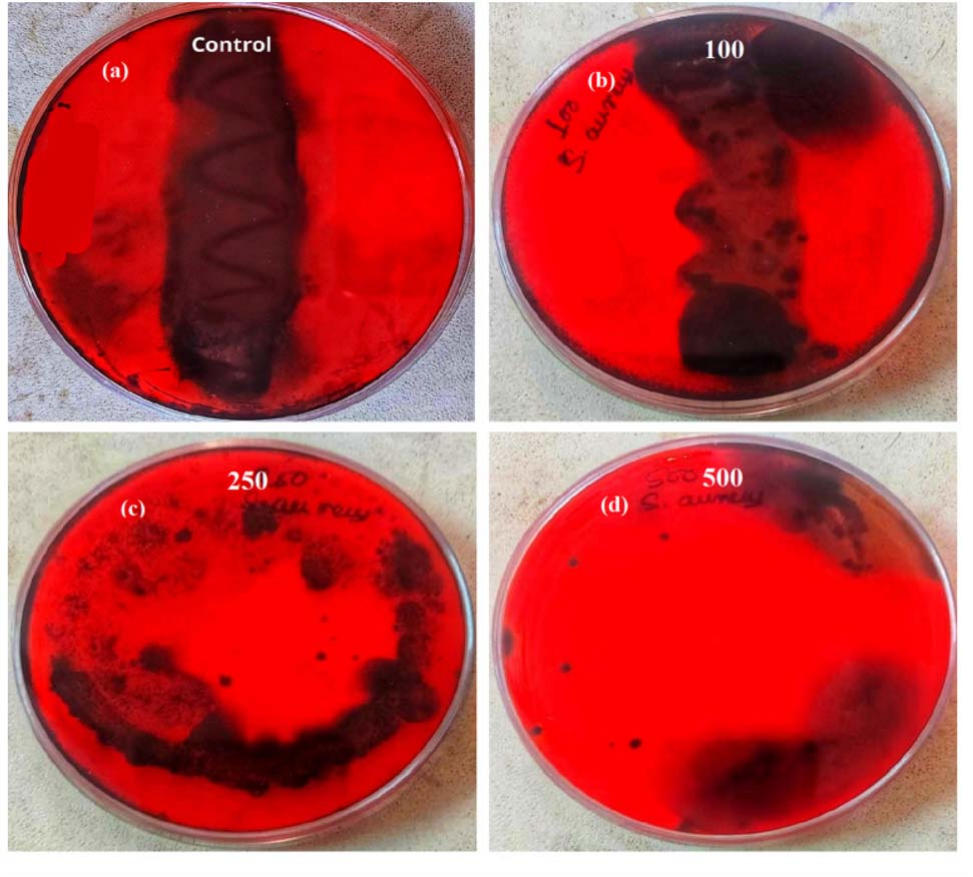
Anti-biofilm activity of *S. aureus* in absence (a) and presence of different concentrations of SeNPs such as (b) 100 μg mL^−1^, (c) 250 μg mL^−1^, (d) 500 μg mL^−1^ respectively. Black dry colonies formed on the agar plate indicate the biofilm activity of *S. aureus* and reduction in the formation of black colonies upon treatment of different concentrations of SeNPs indicates the anti-biofilm activity of SeNPs.

**Fig. 4:**
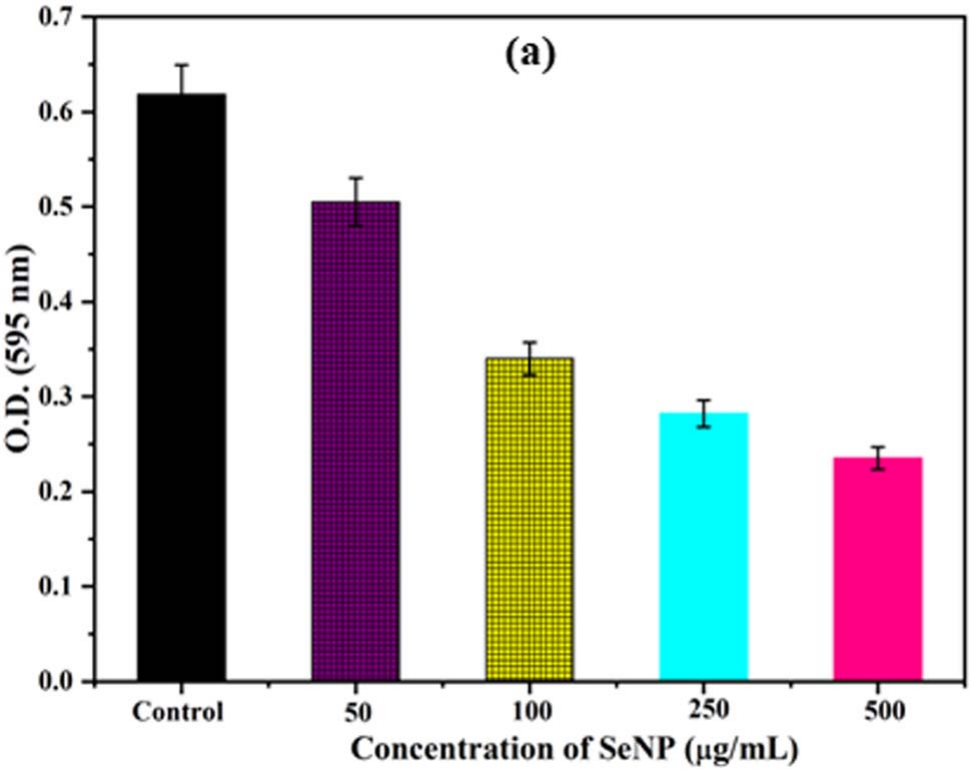
Anti-biofim activities of *S. aureus* upon treatment of different concentrations of SeNPs.

Biofilm formation by bacteria play key role in determining the pathogenicity of the bacteria. Initially, the anti-biofilm activity of biosynthesized SeNPs was analysed against *S. aureus* using Congo red agar plate method. From the Fig. 3a, it was noted that black colonies were formed on Congo red agar plate in the absence of SeNPs, which interpret the formation of biofilm. However, in presence of different concentrations of SeNPs (100, 250 and 500 μg mL^−1^), we observed gradual decrease in black colour colony formation, indicating the anti-biofilm activity of SeNPs (Fig. 3).The anti-biofilm activity of biosynthesized SeNPs was further evaluated against *S. aureus* using microtiter plate technique. The bacterial strains without treatment of SeNPs (control) exhibit higher optical density indicating strong biofilm formation (Fig. 4). However, upon increasing the concentrations of SeNPs, we observed decrease in optical density of bacterial culture, indicating decrease in biofilm formation (Fig. 4). Additionally, we observed that higher concentration of SeNPs such as 500 μg mL^−1^ exhibits strong anti-biofilm activity against the test bacteria.

### 3.5 Photocatalytic activity

**Fig. 5:**
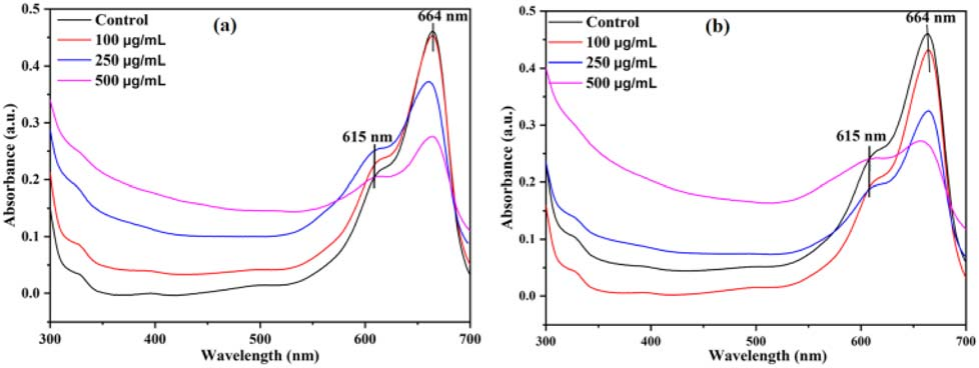
Photodegradation of methylene blue upon treatment with SeNPs after 0.5 h (a) and 2 h (b) respectively.

It was reported that different NPs with photocatalytic activity are able to generate ROS upon interaction with sunlight irradiation, hence exhibiting both antimicrobial and anti-biofilm activities. The photocatalytic activity of SeNPs biosynthesized from *S. aureus* was analysed using the degradation of methylene blue (MB), an organic dye, under solar irradiation. Upon addition of SeNPs, after couple of hours the deep blue colour of MB changed into light blue colour. As reported, in the UV-Visible spectrum, methylene blue generally gives rise to two absorption peaks at 664 nm and 619 nm. However, for estimating the photocatalytic activity, among two peaks, peak at 664 nm is taken as reference.^29^ In our study, the absorbance of MB was taken from 300 nm to 700 nm and the absorption peaks for MB was observed at 664 nm and 615 nm (Fig. 5). As anticipated, the intensity of absorption peak at 664 decreased upon addition of different concentrations (100, 250 and 500 μg mL^−1^) of SeNPs at different time intervals. Additionally, we observed that the photocatalytic activity of SeNPs increases with increasing the concentrations SeNPs.

Selenium, acting as a mild oxidizing agent, belongs to group 16 (oxygen family) in the periodic table. It has been reported that, in suspension, MB acts as a cationic agent.^30^ To make the electron transfer process less complicated in oxidation–reduction reactions, an effective catalyst should have intermediate redox potential value between acceptor and donor.^29^ It has been reported that, SeNPs acts as a catalyst. When the reaction between SeNPs and MB occurs, Se donates an electron to methylene blue that ensures reduction of MB into leuco MB. As a result, colourless or lecuo methylene is formed that lead to the hypochromic shift of absorption peak from 664 nm to 615 nm (Fig. 5).

### 3.6 Study on ROS detection

**Fig. 6:**
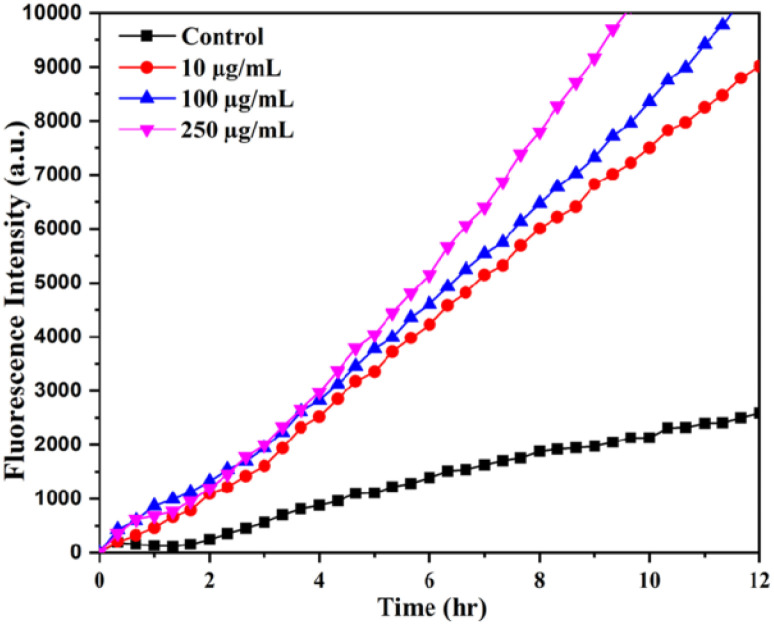
The ROS-specific fluorescent dye (DCFH-DA) intensity at different time intervals upon the biosynthesized SeNPs treatments with *E. coli*.

Fig. 6 shows the kinetics of DCFH-DA dye oxidation in *E. coli* cells upon treatment with different concentrations of SeNPs such as 10, 100 and 250 μg mL^−1^. Increase in fluorescence intensity was observed with increasing the concentration of SeNPs. DCFH-DA is a peroxynitrite indicator used for the detection of ROS formation inside the cell.^18^ It was observed from the figure that ROS is also produced in the culture media to some extent in the absence of SeNPs. However, ROS produced in non-stress condition is counterbalanced by ROS scavenging enzymes. Upon increasing the concentration of SeNPs, it was observed that fluorescence intensity of DCFH-DA increases many folds, indicating the formation of ROS. It was also noted that the production of ROS increases with the increase in time period upon addition of SeNPs.

The eradication of biofilm is necessary in the present scenario, especially in hospitals in order to curb the development of microbial resistance. Biofilm is defined as organised bacterial communities enclosed in a autogenous extracellular polymeric substance (EPS) such as exopolysaccharide, extracellular DNA (eDNA), and proteins adhered to abiotic or biological surfaces. Bacterial cells in biofilm are better defended, minimally subjected to mutation, more defiant to antibiotics, and represent reduced metabolic activity.^31^ Biofilm and antibiotic resistance have quite a strong relationship and this problem is rarely resolved. Antimicrobial agents are unable to penetrate into biofilm network serving as one of the most vital reasons for the development of resistant microbial strains. Nanostructured materials have shown encouraging results upon applications exhibiting anti-biofilm activity.^32^ SeNPs have the amazing properties to destroy the biofilm formation by the microbes. The molecular mechanisms of biofilm-inhibitory effect for SeNPs and other Se compounds have not been completely understood and needs further investigation. Also literatures suggest that SeNPs induce a higher production of ROS compared to selenite.^33^

ROS stands for reactive oxygen species comprising of peroxide, superoxide anion, hydrogen peroxide, singlet oxygen, hydroxyl ions, and hydroxyl radicals.^34,35^ It has high therapeutic role in inhibiting various microbial biofilms inhabiting skin, intestinal tissues, *etc.*^36^ Within the microbial cell, the environmental oxygen participates in oxidative phosphorylation, reducing the O_2_ to water, thereby generating energy to aerobic microbes. But, when the level of O_2_ surpasses the native habitat O_2_ level, certain biological molecules such as flavoenzymes start interacting with the O_2_ leading to decline in growth, enhanced mutagenesis, and death.^37^ Facultative anaerobic microbes such as *E. coli* are also met with the same fate. The toxicity exhibited by reduced ROS is quite similar to those exhibited by ionizing radiations.^38^ In many microbes, the pyruvate:ferredoxin oxidoreductase and nitrogenase pathways are destroyed which employ low-potential Fe–S clusters to meet their complex metabolic chemistry.^38^ The biosynthetic pathways of amino acids are also disrupted by the O_2_ along with the assistance of their certain ROS species such as hydrogen peroxide, and superoxides. Hydrogen peroxides also target the Fe ions that are left unincorporated in the systems as in the case of DNA thereby leading to DNA damage. The metal nanoparticles can take advantage of their enhanced ROS generation property and employ oxidative stress in microbes that are being targeted to be neutralized in order to serve as clinical therapeutics.^38^

Studies have evidenced that regardless of its dimensions, SeNPs generate higher amount of ROS in comparison to selenite.^39^ This suggests that involvement of ROS in the toxicity of NPs, must be following some intricate mechanisms accountable for the antimicrobial activity of these nanostructure materials. For instance, it seems that NPs can contribute to functional damages of cell membrane or wall by disrupting the integrity of the important building elements and outer envelopes.^40^ It can be inferred that the surface features of the NPs may be involved in conferring toxicity to NPs *via* some other related mechanisms which needed further investigation. Currently, the molecular mechanisms of biofilm-inhibitory effect for SeNPs and other Se compounds demands further studies since they are not completely understood.

### 3.7 Estimation of cytoplasmic leakage

**Fig. 7:**
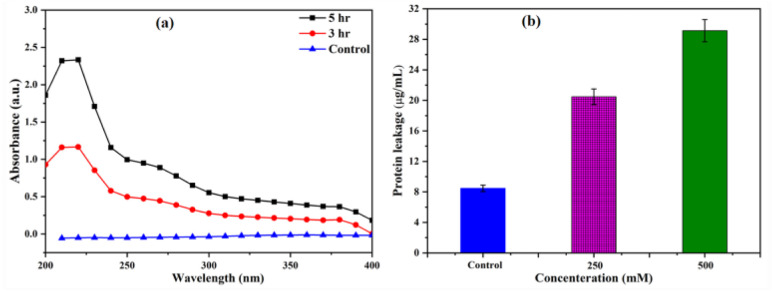
Quantification of cytoplasmic leakage of DNA (a) and protein (b) upon treatment with 250 μg mL^−1^ and 500 μg mL^−1^ of SeNPs in *E. coli* culture. Culture without SeNPs treatment was taken as control.

It has been reported that NPs can affect the bacterial cell membrane adversely leading to leakage of cytoplasmic materials through membrane.^13^ To gain more insight into the effects of SeNPs on bacterial membrane integrity, we quantified the cytoplasmic materials, such as DNA and protein of *E. coli*, upon treatment with SeNPs. Upon centrifugation, the supernatant of both SeNPs treated and untreated bacterial cells were analysed to evaluate the quantity of leakage protein and DNA. As shown in the Fig. 7a, It was observed that, after 3 h of SeNPs treatment the absorbance of bacterial solution was increased at 260 nm in comparison to control, inferring the leakage of cytoplasmic DNA content of test bacteria. Additionally, it was also observed that, the absorbance maxima was further increased after 5 h of treatment, inferring higher amount of cytoplasmic DNA leakage. However, leakage of protein was estimated by Bradford assay upon treatment of SeNPs (Fig. 7b). As shown in the figure, the amount of protein leakage was increased with increase in the concentration of SeNPs in comparison to control. Highest amount of protein leakage was observed at 500 μg mL^−1^ concentration of SeNPs.

Recent studies have reported that due to advance physico-chemical properties, NPs exhibit significant antimicrobial activity against various infectious microorganisms. Among, various NPs, SeNPs have drawn the attention of various research groups for various biomedical applications. SeNPs possess advanced physicochemical properties, including electron transfer capability and high chemical stability, making them suitable for various biomedical applications.^41^ Although, several research groups have already proposed various mechanisms for the antimicrobial activity of SeNPs, the exact mechanism has not been fully explored. Hence, understanding the exact mechanism behind antimicrobial activity of SeNPs is necessary for safe use of SeNPs as novel antimicrobial agent. In this study, we have taken an attempt to explore the mechanism behind antimicrobial activity of SeNPs against Gram-positive and Gram-negative bacteria using various antimicrobial and biophysical techniques. Initially, this study presented a continuous production protocol for the synthesis of SeNPs using *S. aureus* at MIC concentration of sodium selenate. The method can be optimised for large scale production of SeNPs, which will be able to fulfil the need of various nanotechnology industries. As observed from growth kinetics, under normal condition (without treatment of SeNPs) both the bacterial culture exhibited all the phases. However, upon treatment with SeNPs, we observed reduction of log phase for each bacterium, elucidating antimicrobial activity, which was further confirmed by CFU analysis. From the findings, it was suggested that SeNPs have the potential to combat both Gram-positive and Gram-negative bacteria. Gram-negative bacteria are more sensitive towards SeNPs compared to Gram-positive bacteria due to structural and chemical composition of the cell membrane. The outer membrane of Gram-negative bacteria is covered with lipopolysaccharide which is less rigid and easily breakable in comparison to peptidoglycan present in the outer membrane of Gram-positive bacteria.^42^ The evaluation of cytoplasmic leakage of protein and DNA demonstrated that SeNPs interface put stress on bacterial cell membrane causing leakage of cytoplasmic contents. The study also evaluated the photocatalytic activity of biosynthesized SeNPs and the amount of ROS generated due to photocatalytic activity of SeNPs. From the experimental data we hypothesized that ROS generated at SeNPs-bacteria interface put stress on bacterial membrane causing leakage of cytoplasmic materials leading to bacterial cell death. In this context, Menon *et al.* have also synthesized SeNPs using extract of cow urine and evaluated the antimicrobial activity against both Gram-positive and Gram negative bacteria.^43^ Additionally, Geoffrion *et al.* have also formulated pure naked selenium nanoparticles using green synthesis method and evaluated the antimicrobial activity. From the findings, they have suggested that SeNPs exhibited antimicrobial activity at very low concentration such as 0.5 to 1 ppm, without exhibiting significant cytotoxicity against human healthy cells.^44^

## 4. Conclusion

From the above findings, it was concluded that the generation of ROS at the SeNPs interface due to photocatalytic activity of biosynthesized SeNPs plays an important role in the determination of antimicrobial activity. SeNPs was biosynthesized from *S. aureus* using the principle of green chemistry and characterized using various biophysical techniques. The biosynthesized SeNPs showed strong antimicrobial activity against both *B. subtilis* and *E. coli* and anti-biofilm activity against *S. aureus*. From the above study, it was observed that, photocatalytic activity increases with increase in concentrations of SeNPs leading to higher ROS formation; hence increase in antimicrobial and anti-biofilm activity upon increasing the concentrations of SeNPs. The ROS generated at the SeNPs interface put stress on the bacterial cell membrane leading to leakage of cytoplasmic materials of the bacterial cells resulting in non-viable bacterial cells. As a conclusion, the above study optimised a novel protocol to formulate SeNPs at MIC concentration of sodium selenate against *S. aureus*, which would be able to fulfil NP-mediated industrial and theranostics applications. Additionally, the above formulation could be adopted as a potential antimicrobial formulation against various bacterial strains.

## Author contributions

Manoranjan Arakha: conceptualization, formal analysis, methodology, supervision, writing original draft, review & editing, Banishree Sahoo: conceptualization, investigation, methodology, validation, writing original draft, Lipsa Leena Panigrahi: writing original draft, Sonali Jena: data curation, Suman Jha: review & editing.

## Conflicts of interest

The authors have declared that they have no conflict of interest.

## Supplementary Material
